# Inhibition of cyclin dependent kinase 9 by dinaciclib suppresses cyclin B1 expression and tumor growth in triple negative breast cancer

**DOI:** 10.18632/oncotarget.10870

**Published:** 2016-07-28

**Authors:** Sandeep Rajput, Nimmish Khera, Zhanfang Guo, Jeremy Hoog, Shunqiang Li, Cynthia X. Ma

**Affiliations:** ^1^ Section of Medical Oncology, Division of Oncology, Department of Internal Medicine, Washington University School of Medicine, St. Louis, MO, USA; ^2^ Siteman Cancer Center, Washington University School of Medicine, St. Louis, MO, USA

**Keywords:** dinaciclib, triple negative breast cancer, patient derived xenograft, cyclin B1, cyclin dependent kinase 9

## Abstract

Cyclin-dependent kinases (CDKs) are potential cancer therapeutic targets because of their critical role in promoting cell growth. Dinaciclib is a novel CDK inhibitor currently under clinical evaluation for the treatment of advanced malignancies. In this study, we demonstrated the anti-tumor activity of dinaciclib in triple negative breast cancer (TNBC) patient derived xenograft (PDX) and cell lines *in vitro* and *in vivo*. Treatment with dinaciclib induced cell cycle arrest at G2/M phase and marked apoptosis. These changes were accompanied by reduced phosphorylation of CDK1 and retinoblastoma (Rb) protein and decreased protein levels of cyclin B1, cMYC and survivin. We further demonstrated that siRNA knockdown of CDK9, the kinase subunit of positive transcription elongation factor b (P-TEFb), instead of CDK1 or CDK2, reduced the levels of cyclin B1 and MYC in TNBC cell lines. These data support the importance of CDK9, in addition to CDK1, in mediating the growth inhibitory effect of dinaciclib in TNBC. Further investigation of CDK9 as a therapeutic target in TNBC is needed.

## INTRODUCTION

Triple negative breast cancer (TNBC) refers to the subgroup of breast cancers that are negative for estrogen receptor (ER), progesterone receptor (PR), and HER2. It accounts for about 15% of invasive breast cancer diagnosis and is often associated with high tumor grade, aggressive clinical course, and adverse prognosis [1]. Compared to the availability of targeted agents for ER+ or HER2+ breast cancer, treatment for TNBC is limited to cytotoxic chemotherapy which is often ineffective, leading to recurrence and death [1, 2]. Development of molecularly targeted agents is therefore in great need [2].

A major advance in recent years is the improved understanding of the genetic basis of TNBC through genome-wide investigations including The Cancer Genome Atlas (TCGA) and others [3–5]. A prominent finding in TNBC from these studies was the lack of common driver mutations identified in ER+ or HER2+ disease, and the loss of key regulators of cell cycle checkpoints, including mutations in *TP53* (84%), loss/mutations of *RB1* (20%) and germline and/or somatic mutations in *BRCA1* or *BRCA2* (20%), as well as gain of cell cycle promoting genes such as amplifications of *CCNE1* (9%) and *c-MYC* (40%), among others [3]. Therefore targeting cell cycle regulatory proteins presents an attractive therapeutic strategy in TNBC [6–8].

Cyclin dependent kinases (CDKs) are key positive regulators of cell cycle progression, transcription, and mRNA processing [6, 8]. Inhibitors against CDK4/6, which induce G1 to S phase cell cycle arrest, have demonstrated therapeutic efficacy in both endocrine therapy naïve and resistant ER+ breast cancer [9–11]. Palbociclib, the first selective CDK4/6 inhibitor introduced in the clinic has received FDA approval to combine with letrozole as first line therapy and to combine with fulvestrant after disease progression on prior hormonal therapy for patients with metastatic ER+ HER2- breast cancer [10–12]. However, TNBC is often resistant to CDK4/6 inhibition in preclinical studies [9, 13], likely as a result of deficiency in RB [3]. In contrast, inhibitors against other CDKs have shown promising activity [8, 14].

Dinaciclib is a novel, potent, small molecule inhibitor against CDK1, CDK2, CDK5, and CDK9, with IC50 values of 3, 1, 1, and 4 nmol/L, respectively [15]. Compared with flavopiridol, the first CDKs inhibitor to enter in clinical trials, dinaciclib exhibited superior activity with a better side effects profile [15]. Dinaciclib has shown promising antitumor activity in preclinical studies in broad spectrum of cell lines with median IC_50_ of 11nM and in early phase clinical trials of hematological and solid malignancies including breast cancer [16–27]. In cell line studies, dinaciclib was found to be particularly effective in TNBC with elevated MYC [14]. However the molecular mechanisms underlying the sensitivity of dinaciclib in TNBC are not fully understood. In this study, we aimed to investigate the activity of dinaciclib in patient derived xenograft models and to further investigate the mechanisms of action of dinaciclib to uncover novel therapeutic targets in TNBC. We demonstrated that dinaciclib was highly effective in inhibiting tumor growth in TNBC PDXs *in vitro* and *in vivo*. Importantly, we demonstrated a reduction in the protein levels of cyclin B1 and MYC upon dinaciclib treatment through inhibition of CDK9, suggesting CDK9, MYC and cyclin B1 as potential therapeutic targets in TNBC.

## RESULTS

### Dinaciclib inhibited TNBC cell growth *in vitro*

**Figure 1:**
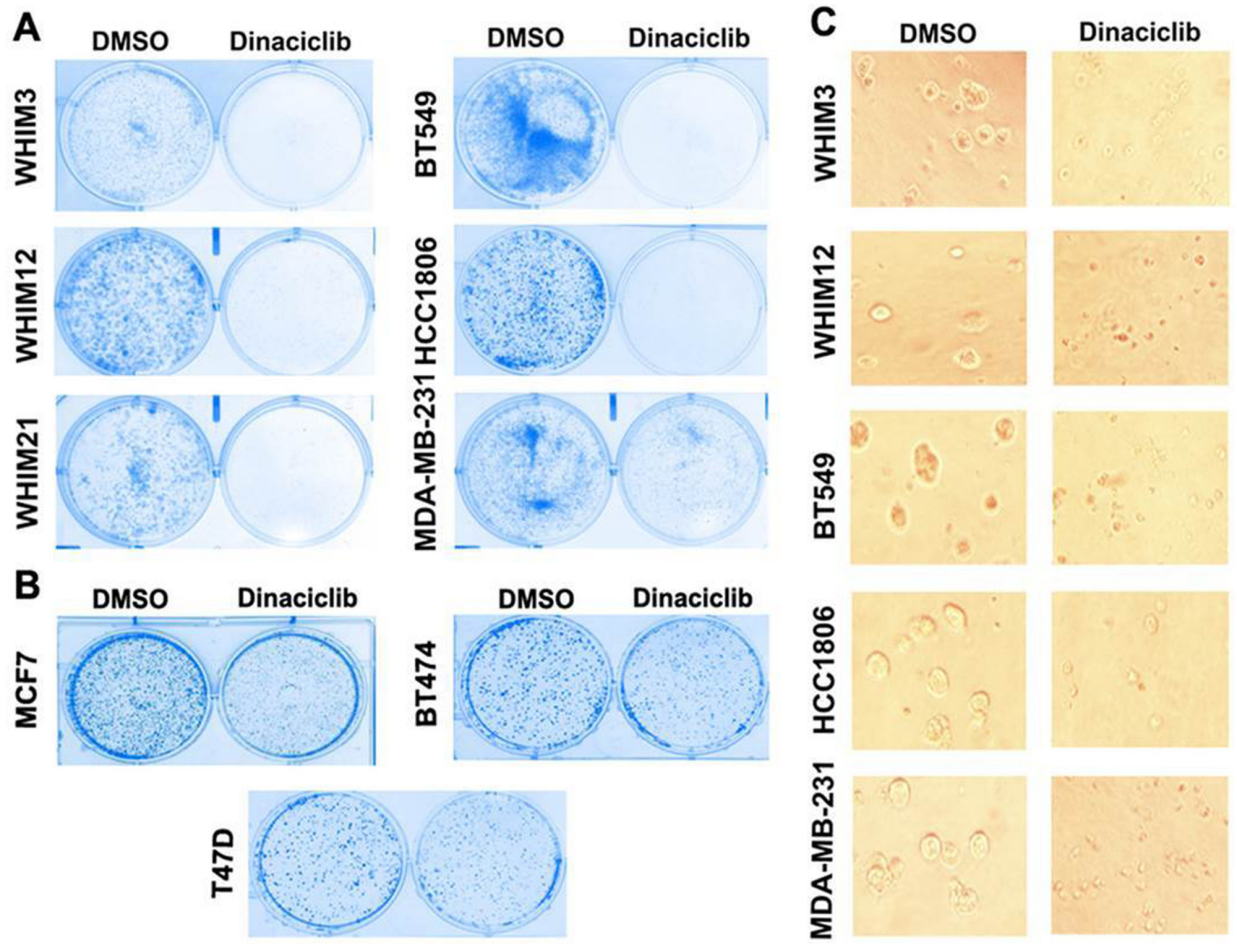
Dinaciclib inhibits cell proliferation of TNBC more effectively than that of ER+ breast cancer **A.** Colony formation assay of TNBC from PDXs (WHIM3, WHIM12, and WHIM21) and cell lines (BT549, HCC1806, MDA-MB-231) in the presence of DMSO or 10mM dinaciclib. **B.** Colony formation assay of ER+ breast cancer cell lines (BT474, MCF7 and T47D) in the presence of DMSO or 10nM dinaciclib. **C.** Three-D matrigel assay of TNBC from PDXs (WHIM3, WHIM12, and WHIM21) and cell lines (BT549, HCC1806, MDA-MB-231) in the presence of DMSO or 10nM dinaciclib.

We first performed 2D clonogenic assay to determine the effect of dinaciclib on *in vitro* cell growth of 3 PDX models as described in materials and methods [WHIM3 (WT *TP53*), WHIM12 (mutant *TP53*) and WHIM21 (mutant *TP53*)], 3 TNBC cell lines [BT549 (mutant *TP53*), MDA-MB-231 (mutant *TP53*), and HCC1806 (mutant *TP53*), and 3 ER+ cell lines [BT474 (WT *TP53*), MCF7 (WT *TP53*) and T47D (mutant *TP53*)]. Dinaciclib at 10nM concentration significantly inhibited the colony formation of all TNBC models (Figure 1A). In contrast dinaciclib only partially inhibited the colony formation of ER+ cell lines (Figure 1B), suggesting that TNBC may be particularly sensitive to the growth inhibitory effect of dinaciclib. The growth inhibitory effect of dinaciclib on TNBC was further confirmed in the 3D matrigel assay (Figure 1C).

### Dinaciclib induced apoptosis in TNBC

**Figure 2:**
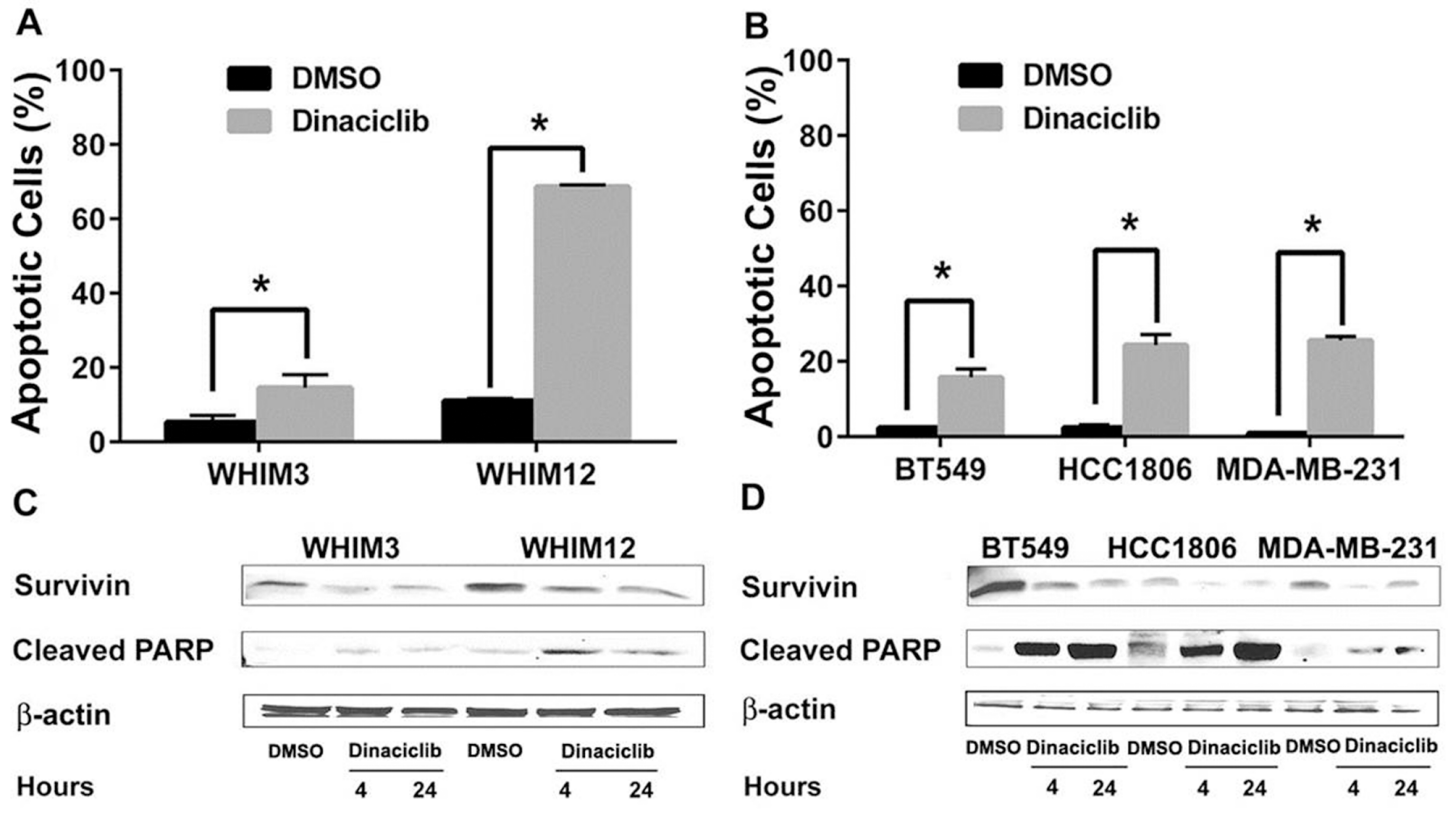
Dinaciclib induced apoptosis in TNBC from PDXs and cell lines. **A.** and **B.** FACS analysis of TNBC cells from PDXs (WHIM3 and WHIM12) and cell lines (BT549, HCC1806, MDA-MB-231) treated with dinaciclib for 24 hours. Percentage of apoptosis (the ratio of Annexin-V and PI-positive cells) was then calculated. Data are presented as average percentage of apoptotic cells ± SEM from 3 experiments. **C.** and **D.** Western blot analysis of apoptosis associated proteins after dinaciclib treatment for 4 and 24 hours. * p<0.05.

To determine the mechanism of cell growth inhibition in TNBC, we next examined whether dinaciclib was able to induce apoptosis *in vitro*. Cells were treated with 50nM dinaciclib for 24 hours followed by quantification of apoptotic cells identified by double-staining with PI and Annexin V by FACS analysis. Dinaciclib induced apoptosis in all TNBC cells tested, including those from PDX models (Figure 2A and 2B). Interestingly, a more dramatic induction of apoptosis by dinaciclib was observed in WHIM12 compared to WHIM3. This effect could be related to high basal expression levels of Cyclin B1 and CDK1 in WHIM12 as compared to WHIM3 (data not shown). To determine the mechanism of apoptosis induction, we investigated the levels of apoptotic and anti-apoptotic proteins following dinaciclib treatment by western blot. Dinaciclib treatment decreased the levels of the anti-apoptotic protein survivin, which is a known CDK1 target [28, 29] and increased in the levels of the cleaved PARP (Figure 2C and 2D). In contrast, treatment with dinaciclib led to little induction of apoptosis in the ER+ cell lines, including MCF7 and T47D cells (Supplementary Figure S1A). There was no reduction in the levels of survivin nor increase in the levels of cleaved PARP observed in MCF-7 and T47D following dinaciclib treatment (Supplementary Figure S2A).

### Dinaciclib induced G2/M phase cell cycle arrest in TNBC *in vitro*

**Figure 3:**
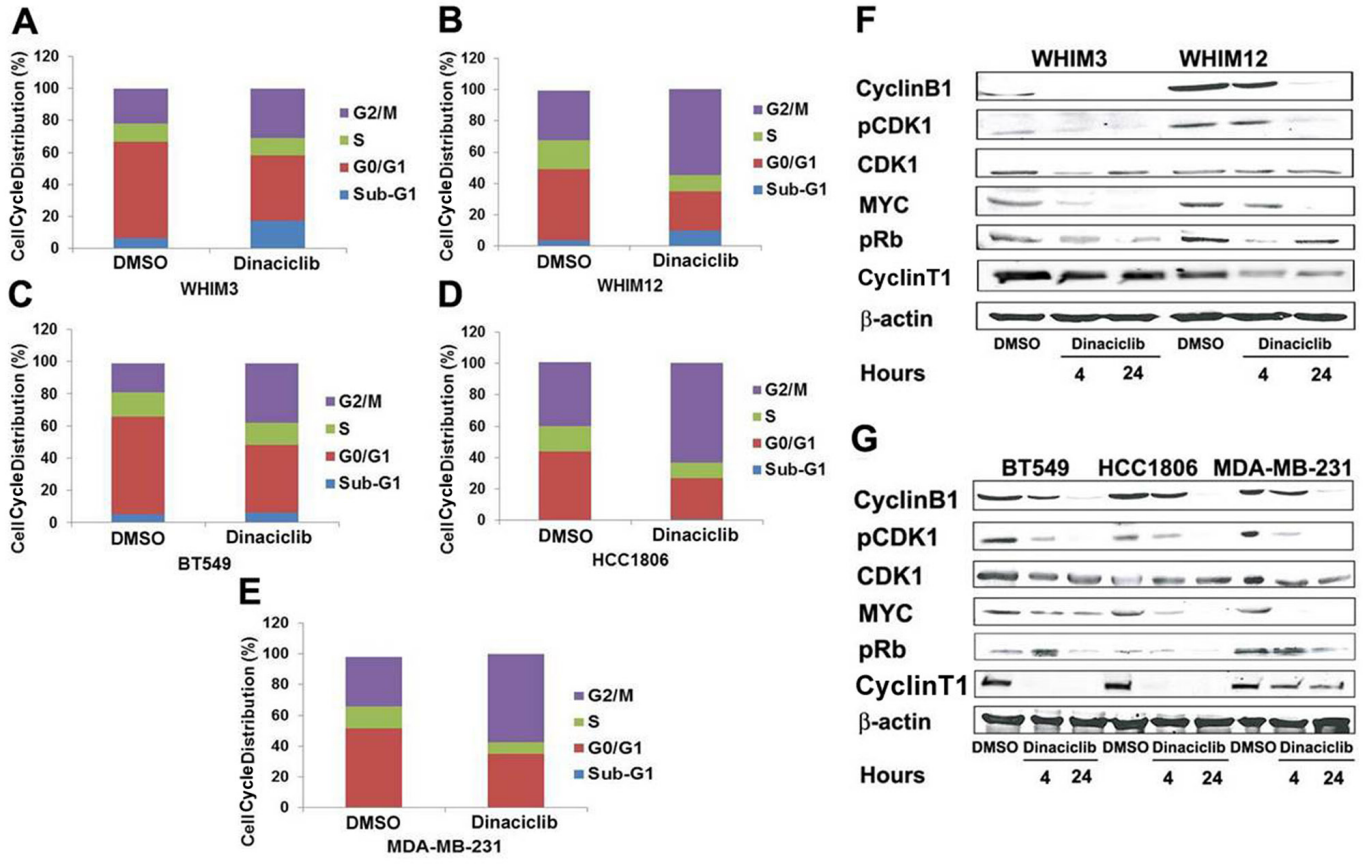
Dinaciclib promotes G2/M phase arrest in TNBC from PDXs and cell lines **A, B, C, D.** and **E.** Cell-cycle analysis by FACS of PI- stained cells treated with dinaciclib for 24 hours. Data in each panel represented the average cell cycle distribution from 3 independent experiments. **F.** and **G.** Western blot analysis of cell-cycle associated proteins after dinaciclib treatment for 4 and 24 hours.

The effect of dinaciclib on cell cycle distribution of TNBC was examined *in vitro*. TNBC cells were treated with 50nM dinaciclib for 24 hours and analyzed by FACS. Dinaciclib treatment led to an accumulation of cells in G2/M phase in all TNBC cell lines tested (Figure 3A, 3B, 3C, 3D and 3E). We then analyzed the protein levels of known cell cycle associated proteins, including CDK1, cyclin B1, Rb and c-MYC [6] by western blot to investigate the mechanisms of cell arrest by dinaciclib. As expected, a significant decrease in pCDK1^T14/15^, and pRb^S807/811^ was observed in all TNBC cell lines following 4-24 hours of exposure to dinaciclib (Figure 3F and 3G). Interestingly, a reduction in the level of cyclinT1 (a binding partner of CDK9), c-MYC and cyclin B1 was also observed post treatment with dinaciclib. Since cyclin B1 is important for CDK1 activation needed for G2 to M phase transition, the reduction in cyclin B1 by dinaciclib could be particularly important for the effect of dinaciclib in TNBC.

### Knockdown of cyclin B1 and CDK1 inhibited cell proliferation in TNBC

**Figure 4:**
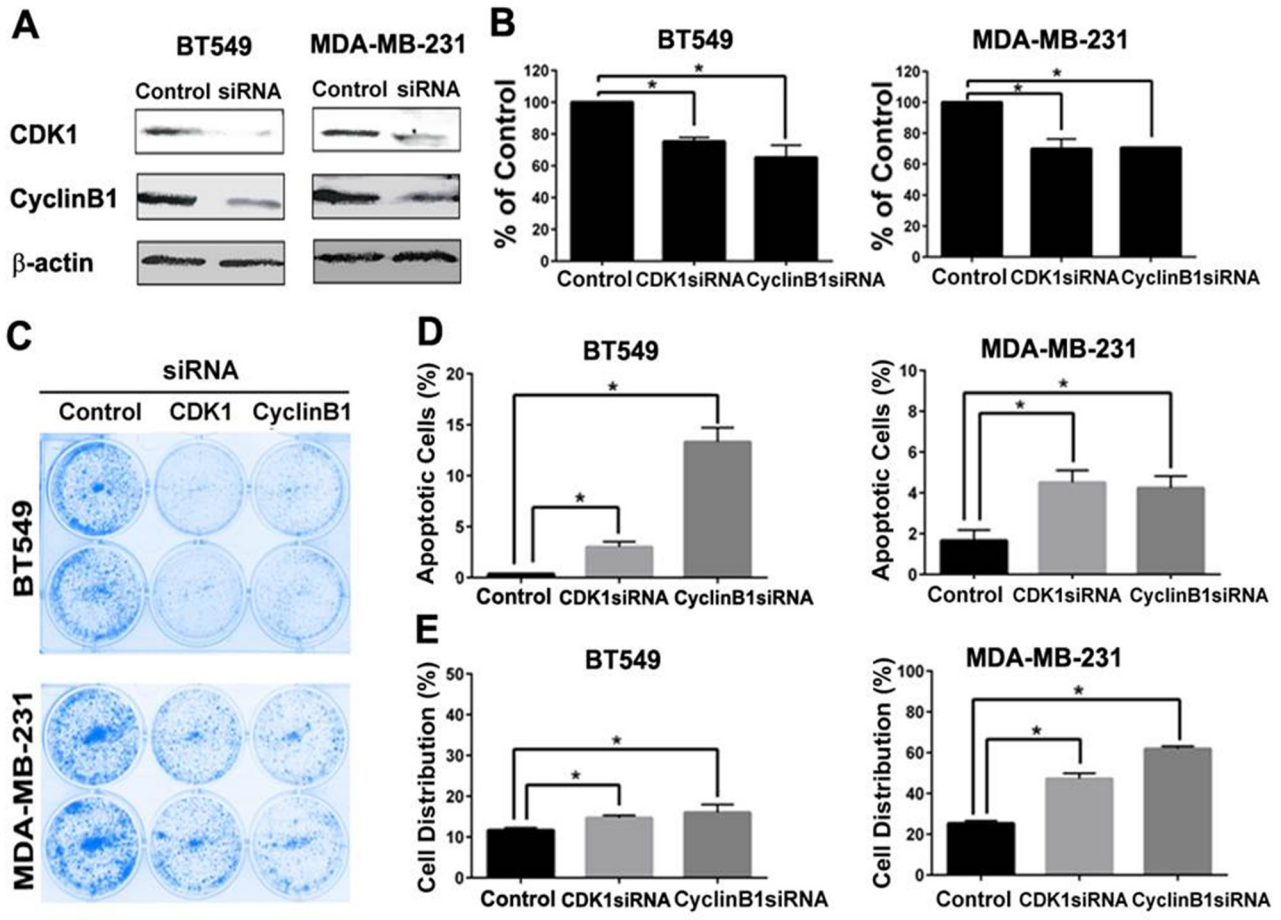
CDK1 or cyclin B1 knockdown inhibited cell proliferation, induced apoptosis and G2/M arrest in TNBC cells **A.** Western blot analysis of CDK1 and cyclin B1 protein level after siRNA knockdown in BT549 and MDA-MB-231. Effect of CDK1 and cyclin B1 knockdown on cell proliferation by Alamar Blue cytotoxic assay **B.** and colony formation **C.** apoptosis induction by Annexin V staining **D.** and G2/M arrest by flow cytometry analysis of cell cycle distribution **E.** were determined. * p<0.05.

Since cyclin B1 in complex with CDK1 drives the progression of cell cycle from G2 to M phase [6], we hypothesized that the inhibitory effect of dinaciclib on CDK1 and cyclin B1 are important for its growth inhibitory effect in TNBC. SiRNA knockdown of CDK1 and cyclin B1 in MDA-MB-231 and BT549 significantly inhibited cell proliferation compared to that of control siRNA as determined by Alamar blue assay (Figure 4B) and colony formation assay (Figure 4C). We next determined the effect of CDK1 and cyclin B1 SiRNA knockdown on cell cycle distribution and apoptosis in BT549 and MDA-MB-231 to determine the mechanism of cell growth inhibition. Both CDK1 and cyclin B1 knockdown promoted significant apoptosis (Figure 4D) and cell cycle arrest in G2/M phase (Figure 4E). These data indicates an important role of cyclin B1 and CDK1 in regulating cell growth and survival in TNBC.

### Dinaciclib targets cyclin B1 through CDK9-MYC axis in breast cancer cells

**Figure 5:**
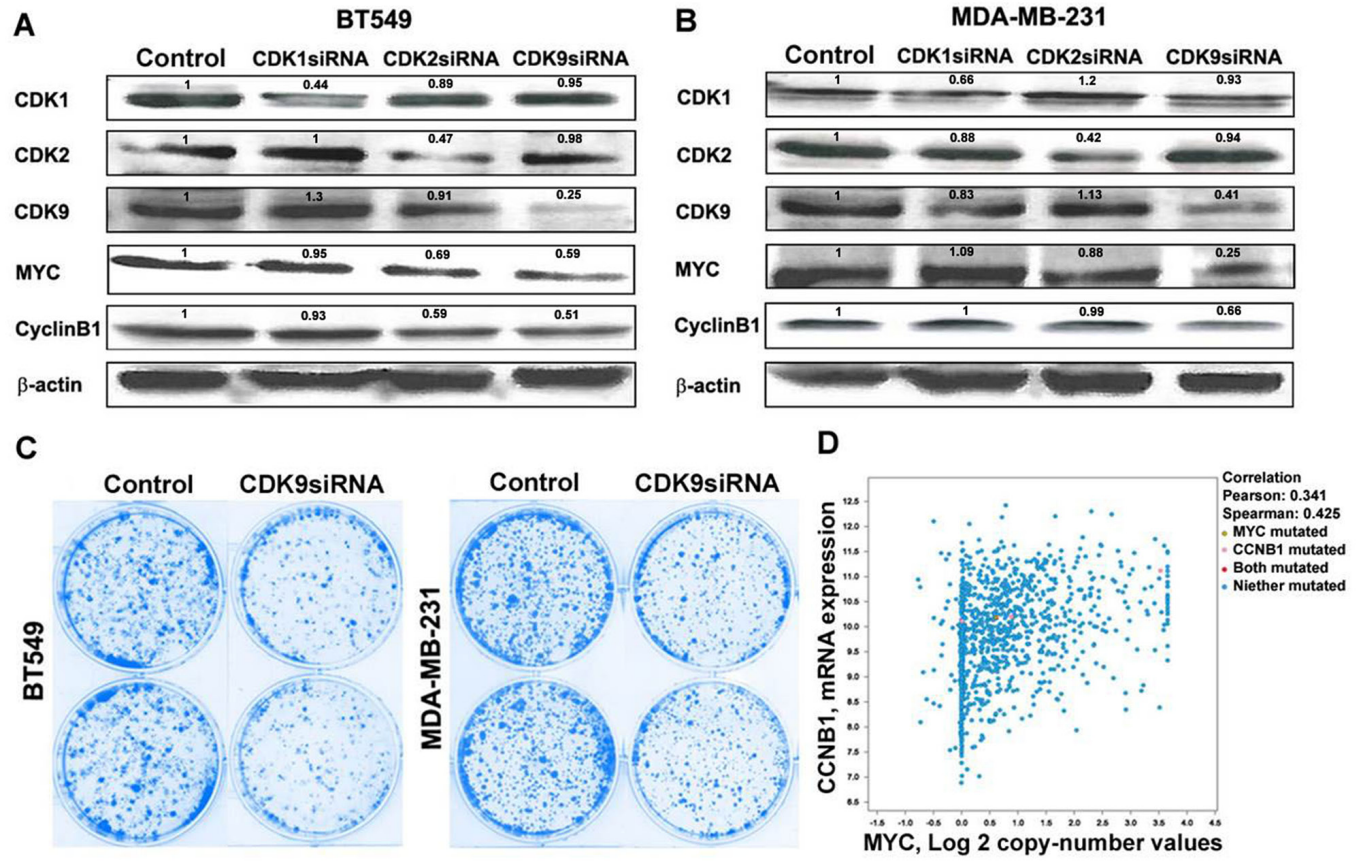
Dinaciclib inhibits cyclin B1 via targeting CDK9-cMYC axis Western blot analysis of CDK1, CDK2, and CDK9 protein levels after siRNA knockdown in **A.** BT549 and **B.** MDA-MB-231 and the effect of CDK1, CDK2, and CDK9 knockdown on cMYC and cyclin B1 protein levels in BT549 and MDA-MB-231 cells. **C.** Effect of CDK9 knockdown on colony formation in BT549 and MDA-MB-231 cells. **D.** Correlation between cyclin B1 mRNA expression level and cMYC copy number in clinical breast cancer patients.

We next investigated the mechanisms of cyclin B1 inhibition by dinaciclib. Since cyclin B1 is a direct transcriptional target of MYC [30] and CDK9, a key component of the positive transcription elongation factor b (P-TEFb) complex [8], required for MYC transcriptional activation of target genes, including MYC itself [26, 31], we hypothesized that dinaciclib reduced cyclin B1 protein expression via CDK9 inhibition in TNBC. As shown in Figure 5A and 5B, knockdown of CDK9, instead of CDK1 or CDK2, by RNAi reduced the levels of cyclin B1 in both BT549 and MDA-MB-231 cells. In addition, knockdown of CDK9 inhibited colony formation in BT549 and MDA-MB-231 cells (Figure 5C), suggesting its potential as a therapeutic target in TNBC. The correlation between cyclin B1 and MYC gene copy number was also observed in clinical breast cancer samples, supporting the role of MYC in regulating cyclin B1 expression (Figure 5D).

### Dinaciclib inhibited tumor growth in TNBC patient derived xenografts tumor model *in vivo*

**Figure 6:**
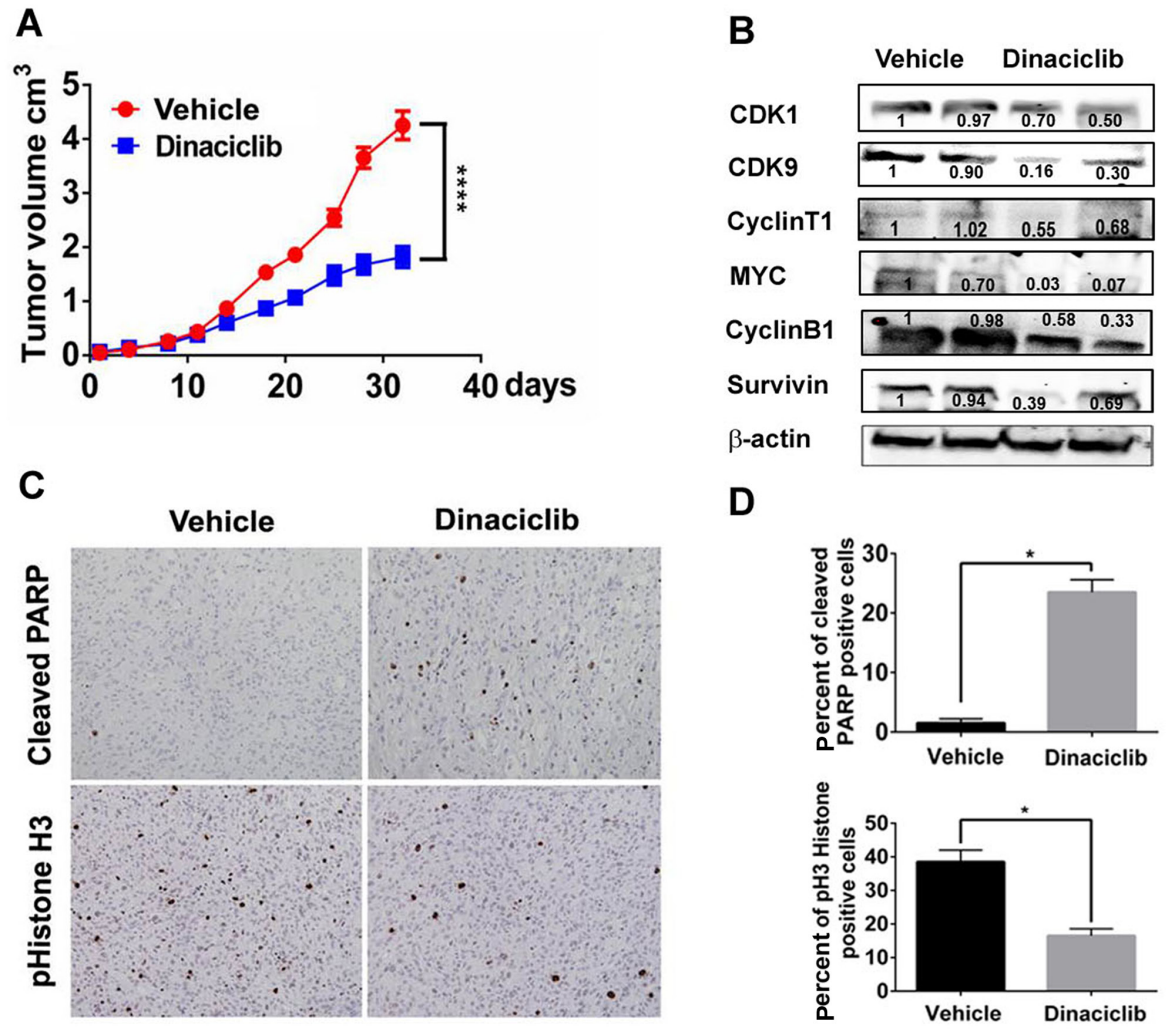
Dinaciclib inhibits tumor growth in a TNBC PDX model *in vivo* **A.** Tumor growth curve of mice bearing WHIM12 PDX treated with vehicle or dinaciclib monotherapy. **B.** Tumor lysates of WHIM12 from vehicle and dinaciclib treated mice were subjected to Western blot analysis of indicated markers. **C.** Immunohistochemistry analysis for phospho-Histone H3, a marker for mitosis on tumor tissue obtained from vehicle and dinaciclib treated mice. **D.** Quantification of the percentage of tumor cells staining positive for cleaved PARP and phospho-Histone H3. * p<0.05

The therapeutic efficacy of dinaciclib was further investigated in TNBC PDX models *in vivo*. TNBC WHIM12 PDX model was chosen because of its particular sensitivity for apoptotic induction by dinaciclib *in vitro*. Dinaciclib or vehicle was administered daily i.p. at 50 mg/kg, days 1-5 each week for 4 weeks in mice bearing WHIM 12 (n=6 each treatment group). A significant inhibition in tumor growth was observed (Figure 6A). At the end of the experiments, tumors were harvested for biomarker analysis. Dinaciclib significantly inhibited the cyclinT1, CDK9, c-MYC, cyclin B1 and survivin protein expression levels in tumor tissues (Figure 6B, n=3). In addition, dinaciclib treatment reduced mitotic entry assessed by IHC of phosphorylated Histone H3, a marker of mitosis, and increased apoptosis assessed by IHC of cleaved PARP significantly (Figure 6D).

### Cyclin B1 gene expression levels negatively correlated with overall survival rate in ER negative breast cancer

**Figure 7:**
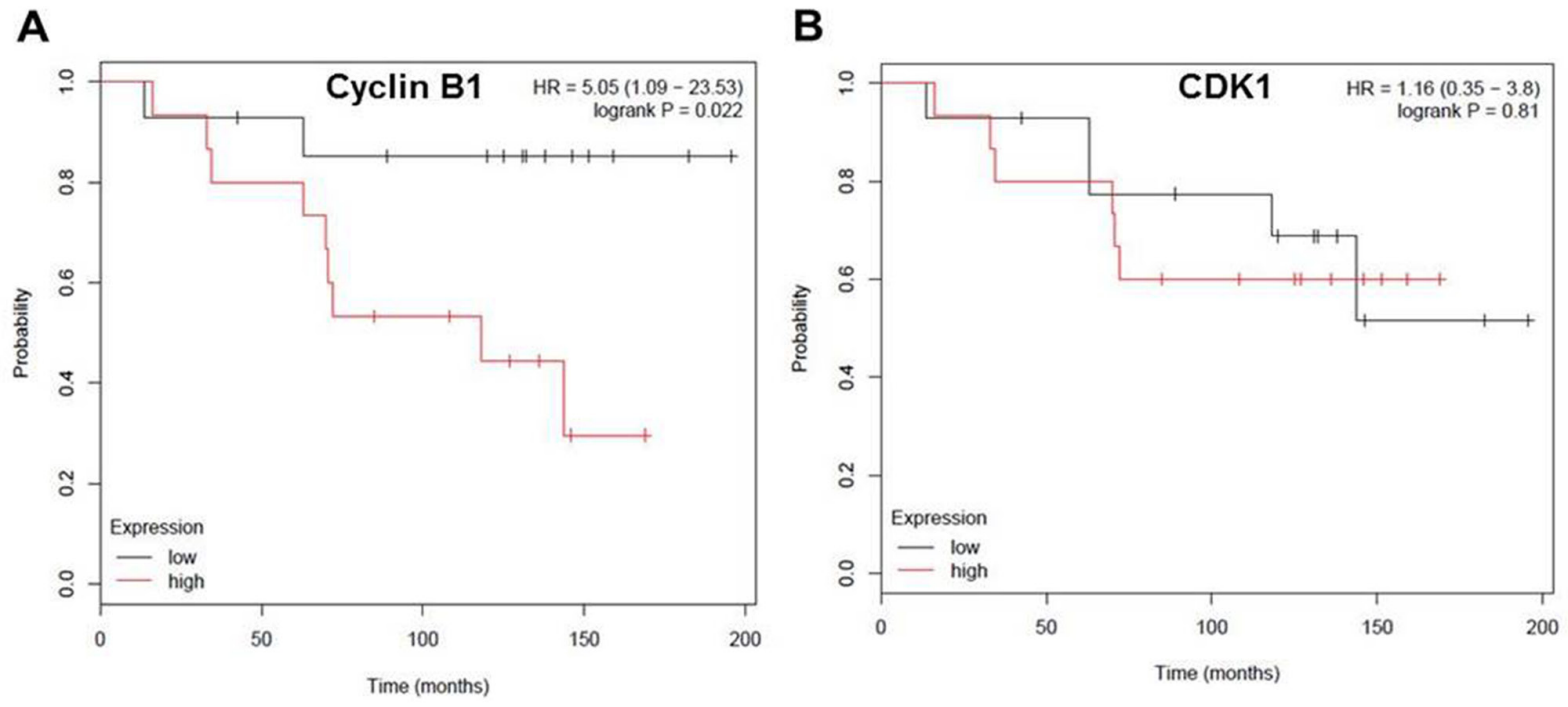
Cyclin B1 expression level negatively correlates with overall-survival rate in patients with ER negative breast cancer **A.** Kaplan-Meier survival curve indicating the correlation between CDK1 expression and overall-survival in chemotherapy treated ER- breast cancer patients. **B.** Kaplan-Meier survival curve indicating the correlation between cyclin B1 expression and overall-survival in chemotherapy treated ER- breast cancer patients.

Next we examined the correlation between expression levels of cyclin B1, CDK1 and overall survival in ER-negative breast cancer patients. We used the on-line tool (www.kmplot.com) which has a combined data set from various annotated breast cancer studies [27]. The Kaplan–Meier plots were generated by the mRNA expression levels of Cyclin B1 and CDK1 for OS of breast cancer patients who received adjuvant chemotherapy. We observed that cyclin B1 gene expression levels negatively correlated with overall survival (OS) in patients treated with adjuvant chemotherapy. (Figure 7A). But there was no significant correlation observed between CDK1 gene expression and OS in these patients (Figure 7B).

## DISCUSSION

CDKs play critical roles in regulating cell cycle transition and gene transcription, which are frequently altered in cancer [32]. Therefore CDKs have attracted much attention in recent years as potential cancer therapeutic targets and selective CDK4/6 inhibitors which potently inhibit the G1 to S phase transition have established their role in the clinic for patients with advanced ER+ breast cancer [32]. However CDK4/6 inhibitors are often ineffective in treating TNBC, likely due to loss of Rb in this cancer type. Interestingly, dinaciclib, an inhibitor against CDK1, 2, 5, and 9 (IC_50_ 1-4nM), but with less activity against CDK4, 6, and 7 (IC50 60-100nM), showed encouraging anti-tumor effect in preclinical studies of TNBC with potential selectivity for MYC driven tumors [13, 14]. However, the clinical activity of dinaciclib in unselected patients with metastatic breast cancer has been modest [22], indicating a need to understand its mechanisms of action for the development of more selective CDK inhibitors in TNBC.

TNBC presents a significant clinical challenge because of the limited therapeutic options. Molecularly targeted agents are in great need, but the development of these agents has been challenging. Unlike ER+ and HER2+ breast cancer, mutations in TNBC often occur in tumor suppressor genes such as *TP53*, *RB1*, and *PTEN* [3], which are not readily targetable. The presence of amplification and/or overexpression of the *MYC* oncogene in TNBC offer an exciting therapeutic opportunity. However, being a transcription factor, MYC targeting with small molecule inhibitors has been difficult to design and develop [33]. Our data suggests the possibility of targeting CDK9 as a strategy for MYC driven TNBC.

Our study demonstrates that dinaciclib, a serine/threonine kinase inhibitor against CDK1, 2, 5, and 9, inhibited tumor growth in TNBC PDXs and cell line models *in vitro* and *in vivo*, by arresting cells in the G2-M phase and inducing apoptosis. We demonstrated for the first time that exposure to dinaciclib reduced the expression of MYC and its transcriptional target cyclin B1 in TNBC. To further investigate the underlying molecular mechanisms, we performed RNAi experiments to individually knockdown CDK1, CDK2, and CDK9 and showed that CDK9, but not CDK1 or CDK2, was responsible for reducing the protein levels of MYC and cyclin B1. Although RNAi knockdown of each of the CDKs (CDK1, CDK2, and CDK9) reduced TNBC tumor growth, and likely that dinaciclib exerted its anti-tumor activity in TNBC by inhibiting all the above CDKs, the finding that CDK9 knock down reduced the expression levels of MYC and Cyclin B1, as well as, tumor growth. Our data on CDK9 identifies that CDK9 inhibition is an important mechanism the anti-tumor effect of dinaciclib and establishes CDK9 as a potential therapeutic target for MYC driven TNBC.

The importance of the proto-oncogene *MYC* in tumorigenesis has been well established [33–36]. It is a helix-loop-helix leucine zipper transcription factor that regulates the expression of a variety of downstream genes important for cell cycle progression, apoptosis, metabolism and cellular transformation [33, 35, 36]. Previous studies demonstrated that MYC enhances transcriptional output by recruiting the CDK9/P-TEFb complex to specific promoters [37–39], and stimulating transcript elongation via pause release [40, 41]. CDK9 as a therapeutic target for MYC driven cancer was demonstrated in a shRNA library screening in which CDK9 was required for the aberrant proliferation of MYC overexpressing hepatocellular carcinoma cell lines [42]. There was a significant correlation between response to CDK9 inhibition and MYC mRNA expression, and CDK9 inhibition impaired RNA Pol II Ser2 phosphorylation and transcription elongation of *MYC* and MYC target genes. This finding was also observed in cell lines from lung and hematopoietic cancers [26, 42]. Our data, for the first time, demonstrated that CDK9 could be an important therapeutic target in TNBC. Further studies are ongoing to further confirm the synthetic lethal interaction between CDK9 inhibition and MYC overexpression/amplification in TNBC using selective CDK9 inhibitors.

Our study suggests that MYC downstream targets cyclin B1 and CDK1 may be particularly important in controlling the proliferation of TNBC. MYC has been considered the direct regulator of cell cycle progression because of its essential role in regulating the expressions of cyclins, including cyclin B1, CDKs and inhibition of CDK inhibitors [33, 35, 36, 43, 44]. *C-MYC* gain has been observed in 40% of TNBC [3]. MYC activation is a characteristic of basal-like breast cancer, the most common molecular subtype of TNBC and is associated with poor prognosis [14, 45, 46]. We demonstrated that inhibition of CDK1 or cyclin B1 by RNAi to reduced tumor cell proliferation in TNBC. In addition, cyclin B1 overexpression was found to correlate with poor clinical outcomes in our study and in others [47]. This finding is also supported by a previous study demonstrating that CDK1 inhibition but not CDK4/6 or CDK2 was selectively lethal to MYC-dependent breast cancer cells [13]. The relevance of CDK1 and cyclin B1 could also be due to their increased levels of gene expression by mutant p53 [7]. Our data also suggest that TNBC may be particularly sensitive to agents that target the G2 to M phase transition.

The effect of dinaciclib on MYC level through inhibition of CDK9 in MYC-driven tumor cells was reported previously in a study of B cell lymphoma cell lines [26]. Although dinaciclib was found to have a preferential growth inhibitory effect in MYC overexpressing TNBC cell lines [14], the dependency of MYC overexpressing TNBC on CDK1 was unclear. Our study confirmed the activity of dinaciclib in TNBC using PDX models and importantly, provided an explanation for the sensitivity of TNBC to dinaciclib and indicated that CDK9 inhibition as potential therapeutic strategy for MYC overexpressing TNBC. Selective CDK9 inhibitors are in preclinical and clinical development and impressive anti-tumor activity has been observed in chronic lymphocytic leukemia cell lines [48]. Studies of selective CDK9 inhibitors in TNBC are warranted. In addition, inhibitors against CDK7, which also plays an important role in phosphorylating the C-terminal domain of RNA polymerase II [49–51] and CDK9 [52], are being developed [53] and could also be important to investigate in TNBC.

## MATERIALS AND METHODS

### Chemicals

Dinaciclib were purchased from (ChemieTek and Sigma). Dinaciclib were prepared in stock solution of 10mM in Dimethyl sulphoxide (Sigma) for *in-vitro* experiments.

### *In vitro* clonogenic assay

We selected 2 human TNBC PDX models (WHIM3 and WHIM12) that differed in TP53 status for our study. The WHIM3 and WHIM12 were generated by engrafting the primary breast tumor of a patient with metastatic TNBC into the humanized mammary fat pad of NOD/SCID mice [28, 30]. We also used 3 TNBC cell lines (BT549, HCC1806 and MDA-MB-231) and 3 ER+ cell lines (BT474, MCF7 and T47D) for our study. Cells were cultured in RPMI-1640 medium with 10% FBS at 37°C in an atmosphere of 5% CO_2_ and 95% air as described previously [54]. For clonogenic assay cells were seeded at a density of 1000 cells per well in 6 well plates in RPMI-1640 medium with 10% FBS for 24 hours. Cells were then treated with dinaciclib at 10nM concentration or dimethyl sulphoxide (DMSO) for 6 days. The colonies were fixed with 4% paraformaldehyde and stained with 0.05% crystal violet in methanol.

### Three-Dimensional Culture in Matrigel

Overlay 3D culture of cells in Matrigel was done as described previously [55]. Cells were seeded in growth factor–reduced Matrigel (BD Biosciences) in 8-well chamber slides. Dinaciclib 10nM or DMSO was added at the time of cell seeding. Cell medium were changed every 3 days and cells were imaged by photomicrograph on day 6.

### siRNA knockdown

Cells were seeded at a density of 200,000 cells per well in 6 well plate in RPMI-1640 medium with 10%FBS. Next day cells were transfected with 50nM CDK1, CDK2, CDK9 and cyclinB1 siRNA (Santacruz) using Lipofectamine 2000 (Invitrogen). Cells were harvested after 48 hours of transfection for western analysis and colony formation assay.

### Western blotting

Cells were seeded in a 6-well plate at the density of 0.5 × 10^6^, adhered overnight followed by treatment with dinaciclib at 50nM concentration for 0, 4, and 24 hours. Cells were harvested after 4 and 24 hours and lysed with 100μL buffer containing 50 mmol/L Tris-HCl, pH 7.5, 150 mmol/L NaCl, 2 mmol/L EDTA, 1% Triton, 1 mmol/L phenylmethylsulfonylfluoride, and Protease Inhibitor Cocktail (Sigma) for 20 minutes on ice. Lysates were cleared at 10,000 rpm for 15 minutes, boiled, separated on 12% SDS gels, and transferred to a nitrocellulose membrane followed by overnight incubation with primary antibodies against pCDK1^T14/15^, CDK1, pCDK2^T160^, CDK2, CyclinB1, p21, pRb^Ser807/8011^, Survivin, cMYC, cleaved-PARP, and β-actin. Protein bands were visualized after 1 hour incubation with HRP-conjugated secondary antibodies and development with ECL (GE Healthcare).

### Antibodies

Primary antibodies were rabbit anti-pCDK1^T14/15^, mouse anti-CDK1, rabbit anti-pCDK2^T160^, rabbit anti-CDK2, mouse anti-cMYC, mouse anti-survivin and rabbit anti- β-actin (Santacruz); anti-p21, anti-pRb, and anti-cleaved-PARP (Cell Signaling); mouse anti-cyclinB1 (BD Transduction). Secondary horseradish peroxidase (HRP)-conjugated anti-rabbit and anti-mouse antibodies were from Jackson ImmunoResearch Laboratories.

### Fluorescence-activated cell-sorting analysis of apoptosis and cell cycle

Apoptotic cells were visualized using Annexin V Detection Kit according to the manufacturer's instructions (BD Pharmingen). Briefly, cells were treated with dinaciclib 50nM for 24 hours followed by incubation with fluorescein isothiocyante (FITC)-conjugated Annexin V and propidium iodide (PI) at room temperature for 15 minutes. After 15-minute incubation, samples were analyzed using AccuriC6 flow cytometer (BD Accuri Cytometers) and cell quest software. Cell-cycle phase distribution was determined in 70% alcohol-fixed cells stained with 5ul PI-RNase A staining buffer (Invitrogen) for 30 minutes at room temperature, followed by analysis using AccuriC6 flow cytometer (BD Accuri Cytometers) and cell quest software.

### Animal studies

WHIM12 was characterized previously [56] and passaged in the “humanized” mammary fat pad of nonobese diabetic/severe combined immunodeficient (NOD/SCID) mice. Passage 4-5 xenografts of WHIM12 were used for the therapy experiments described in this study. A total of 1 × 10^6^ tumor cells and 5 × 10^5^ fibroblasts (2.5 × 10^5^ that were exposed to 4 Gy IR and 2.5 × 10^5^ untreated cells) were mixed and added to an equal volume of a 1:1 mixture of Matrigel (BD Biosciences, Cat. No. 354234) and Collagen I (Millipore, Cat. No. 08-115) to each side of the fourth mammary fat pad that has been cleared of any mouse mammary tissues in female NU/J homozygous mice (Jackson Laboratory, Cat. No. 2019) to propagate xenografts for tumor growth experiments. For long term treatment experiments, xenografts were allowed to grow to approximately 0.5 cm in the maximum diameter. Mice were then divided into 2 treatment groups (*n* = 6 mice in each group): Vehicle (vehicle dilutents) and dinaciclib alone was administered by intraperitoneal injection at a dose of 50 mg/kg/day, 5 days a week for total of 4 weeks. Tumor volume was measured in 2 dimensions (length and width) using Traceable Digital Calipers. The following formula was used to calculate tumor volume: tumor volume (cm^3^) = (length × width^2^) × 0.5. For IHC biomarker studies mice bearing WHIM12 were treated with either Vehicle (*n* = 2), and dinaciclib alone (n=2; 50 mg/kg/day, i.p. days 1, 2 and 3). Tumors were harvested 2 hours after day 3 therapy. Each xenograft tumor was cut into 2 pieces with one piece flash frozen for tumor lysate, and the second piece fixed in 10% neutral buffered formalin and embedded in paraffin blocks. All animal studies were carried out using the appropriate NIH animal care, and the animal study protocol was approved by the Animal Studies Committee of Washington University.

### Immunohistochemistry

IHC for pHistone H3 and cleaved PARP were conducted on 5 μm tissue sections from paraffin-embedded tumor as described previously using the EnVision + Single Reagents HRP-Rabbit (Dako) and REAL substrate buffer (REAL DAB + chromogen, Dako)[28]. The primary antibodies and dilutions are as follows: pHistone H3 (Ser 10) antibody (1:200; Millipore), and cleaved PARP antibody (1:200, Cell Signaling).

### Statistical analysis

Statistical analyses were conducted using Graphpad Prism software. Results are expressed as mean ± SEM. Statistical significance was determined by Student paired *t* test for *in vitro* data respectively. Tumor volume data were compared using one-way ANOVA. *P* ≤ 0.05 was considered significant.

## SUPPLEMENTARY MATERIALS FIGURE


