# Supplementary material for: Inhibition of cyclin dependent kinase 9 by dinaciclib suppresses cyclin B1 expression and tumor growth in triple negative breast cancer
Source: Oncotarget. 2016 Jul 28;7(35):56864–75. doi: 10.18632/oncotarget.10870 (PMC5302958; doi:10.18632/oncotarget.10870)
Supplement: Supplementary file 1 [file oncotarget-07-56864-s001.pdf]

Inhibition of cyclin dependent kinase 9 by dinaciclib suppresses cyclin B1 expression and tumor growth in triple negative breast cancer

SUPPLEMENTARY FIGURE

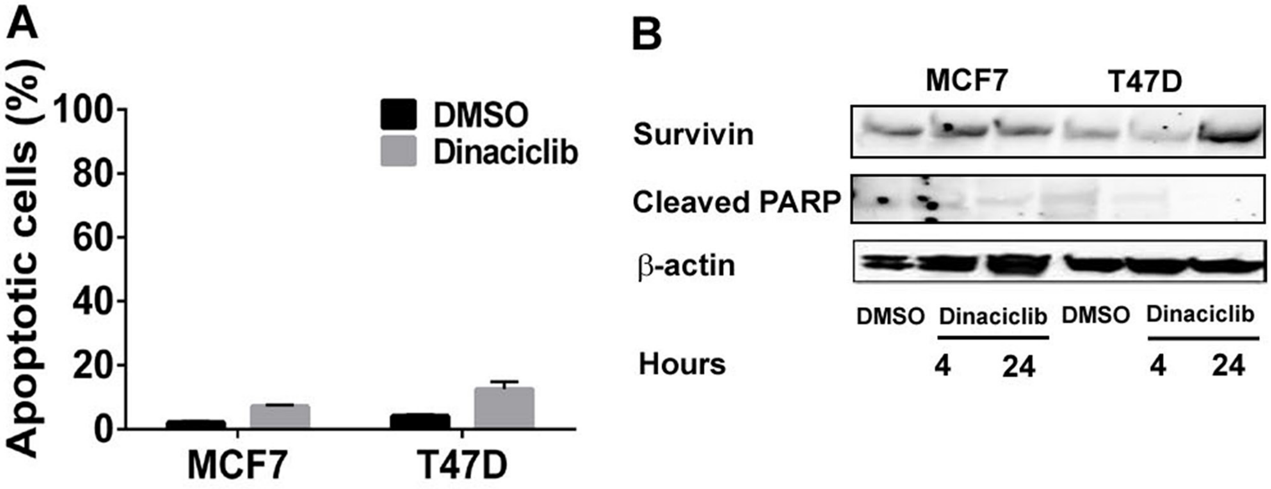

Supplementary Figure S1: Dinaciclib induced very modest apoptosis in ER+ breast cancer cell lines. A. FACS analysis of ER+ breast cancer cells (MCF7 and T47D) treated with dinaciclib for 24 hours. Percentage of apoptosis (the ratio of Annexin-V and PI-positive cells) was then calculated. Data are presented as average percentage of apoptotic cells ± SEM from 2 experiments. **B.** Western blot analysis of apoptosis associated proteins after dinaciclib treatment for 4 and 24 hours.
